# The scaffolding protein AKAP12 regulates mRNA localization and translation

**DOI:** 10.1073/pnas.2320609121

**Published:** 2024-04-23

**Authors:** Madeleine R. Smith, Parisa Naeli, Seyed M. Jafarnejad, Guilherme Costa

**Affiliations:** ^a^Wellcome-Wolfson Institute for Experimental Medicine, Queen’s University, Belfast BT9 7BL, United Kingdom; ^b^The Patrick G Johnston Centre for Cancer Research, Queen’s University, Belfast BT9 7BL, United Kingdom

**Keywords:** mRNA, localization, translation, endothelial, angiogenesis

## Abstract

Regulation of subcellular messenger (m)RNA localization is a fundamental biological mechanism, which adds a spatial dimension to the diverse layers of post-transcriptional control of gene expression. The cellular compartment in which mRNAs are located may define distinct aspects of the encoded proteins, ranging from production rate and complex formation to localized activity. Despite the detailed roles of localized mRNAs that have emerged over the past decades, the identity of factors anchoring mRNAs to subcellular domains remains ill-defined. Here, we used an unbiased method to profile the RNA-bound proteome in migrating endothelial cells (ECs) and discovered that the plasma membrane (PM)–associated scaffolding protein A-kinase anchor protein (AKAP)12 interacts with various mRNAs, including transcripts encoding kinases with Actin remodeling activity. In particular, AKAP12 targets a transcript coding for the kinase Abelson Tyrosine-Protein Kinase 2 (ABL2), which we found to be necessary for adequate filopodia formation and angiogenic sprouting. Moreover, we demonstrate that AKAP12 is necessary for anchoring *ABL2* mRNA to the PM and show that in the absence of AKAP12, the translation efficiency of *ABL2* mRNA is reduced. Altogether, our work identified a unique post-transcriptional function for AKAP12 and sheds light into mechanisms of spatial control of gene expression.

mRNA localization has been described in a multitude of cell types and across taxonomic kingdoms, highlighting its biological significance. Ultimately, this phenomenon supports compartmentalized mechanistic outputs that can respond to local stimuli, orient migration, or even shape cells (1). We revealed that mRNA localization regulates sprouting angiogenesis (2). Sprouting is stimulated by pro-angiogenic factors that activate quiescent vessels and impose dramatic morphological changes in the endothelium (3). The resulting sprout is led by a highly motile and polarized EC, which extends dynamic PM protrusions at the leading edge to sense guidance cues (4). Correct subcellular mRNA localization in these cells underpins EC morphology and behavior during sprouting angiogenesis (2). Here, we set out to identify trans-acting factors in migrating ECs and explore their role in mRNA localization and translation.

## Results

**Fig. 1.**
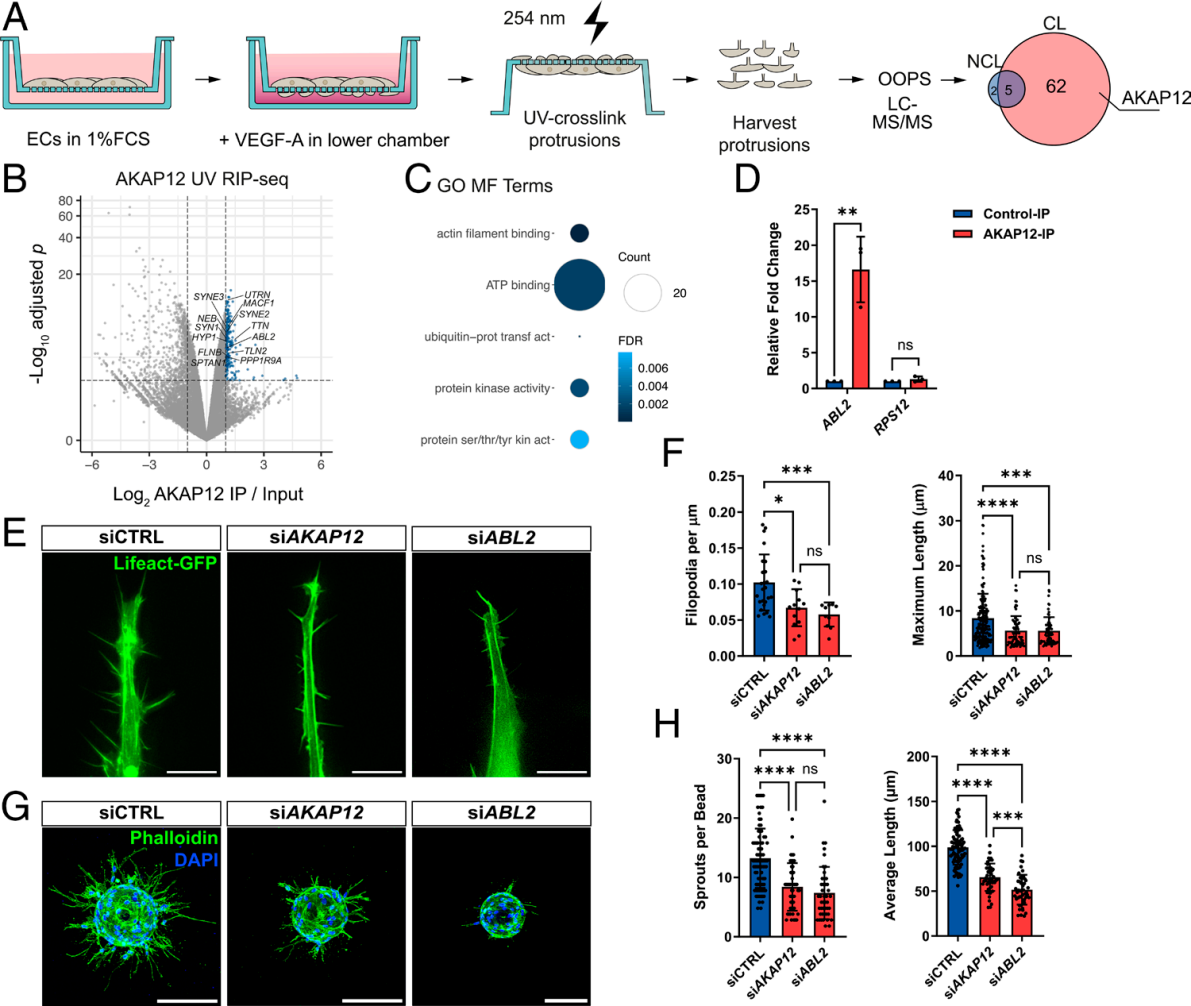
The scaffolding protein AKAP12 binds mRNAs encoding cytoskeletal remodelers. (*A*) Method to isolate and identify RNA-bound proteins in EC protrusions (n = 3). NCL, noncrosslinked; CL, and crosslinked. (*B*) Transcripts identified in AKAP12 UV RIP-seq experiments (n = 3). Labeled dots represent enriched transcripts categorized by Gene Ontology (GO) as Actin filament binding. (*C*) Top 5 GO molecular function categories of transcripts enriched in AKAP12 UV RIP-seq. (*D*) qRT-PCR analysis of *ABL2* and *RPS12* in UV RIP (n = 3). (*E*) Representative ECs cocultured with fibroblasts. (*F*) Quantification of filopodia parameters (*Left*, n = 25 siCTRL, 13 si*AKAP12*, 10 si*ABL2*; *Right*, n = 201 siCTRL, 77 si*AKAP12*, 67 si*ABL2*). (*G*) Representative sprouting beads coated with ECs. (*H*) Quantification of bead assays (n = 84 siCTRL, 48 si*AKAP12*, 45 si*ABL2*). Bars charts represent means ± SD. ns = nonsignificant, * *P* < 0.05, **P < 0.01, ***P < 0.001, ****P < 0.0001. (Scale bars, (*E*) 20 and (*G*) 200 µm.)

To profile the local RNA-bound proteome of EC protrusions, we Ultraviolet (UV)-crosslinked the underside of membranes on which ECs had been briefly cultured and collected protruding material for orthogonal organic phase separation (OOPS) followed by mass spectrometry (Fig. 1*A* and Dataset S1). With this approach, we identified the AKAP12, a member of a family of scaffolding proteins that anchor signaling complexes to cellular microdomains in a spatiotemporal manner (5). Next, to profile the AKAP12-bound transcriptome, we carried out RNA immunoprecipitation (RIP) assays with UV-crosslinked ECs. Subsequent high-throughput RNA sequencing (RNA-seq) revealed 179 transcripts enriched (fold change ≥ 2, adjusted *P* < 0.01) in AKAP12 immunoprecipitates (Fig. 1*B* and Dataset S2), which were strikingly overrepresented in mRNAs encoding kinases and Actin-binding proteins (Fig. 1*C*). Among these, we identified the mRNA encoding ABL2, a nonreceptor tyrosine kinase critical for filamentous (F)-Actin polymerization and PM remodeling (6–8). We focused on this mRNA and validated RNA-seq data with subsequent RIP followed by quantitative (q)RT-PCR. These assays detected high levels of *ABL2* mRNA present in AKAP12 immunoprecipitates when compared to control samples and in striking contrast to the negative control mRNA *RPS12* (Fig. 1*D*).

Due to the implications of both AKAP12 and ABL2 in Actin cytoskeleton biology, we employed siRNA-based loss-of-function assays to examine EC morphogenetic behavior, which could justify the strong association of AKAP12 with mRNA encoding ABL2. First, transfecting ECs with either siRNAs targeting *AKA12* or *ABL2* and cocultured with fibroblasts reduced the number and length of filopodia, thin protrusions rich in F-Actin (Fig. 1 *E* and *F* and Movie S1). Next, using in vitro sprouting assays, which entail dramatic cytoskeletal and PM reorganization (3), we assessed the angiogenic potential of siRNA transfected ECs. Compared to controls, siRNA-transfected ECs generated fewer and considerably shorter sprouting capillaries (Fig. 1 *G* and *H*).

**Fig. 2.**
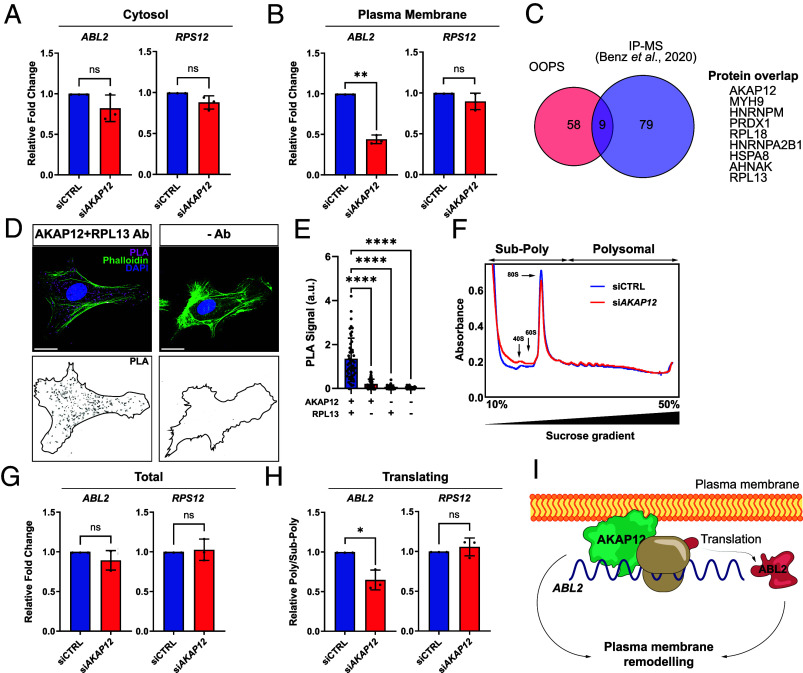
AKAP12 is necessary for efficient *ABL2* mRNA localization and translation. (*A* and *B*) qRT-PCR analysis of *ABL2* and *RPS12* in HEK293 cell fractions (n = 3). (*C*) Overlap of RNA-bound proteins in EC protrusions with AKAP12 coimmunoprecipitating proteins identified in Benz et al. (9). (*D*) Representative PLA performed in ECs. (Scale bars, 20 µm.) (*E*) PLA quantification (left-to-right n = 52, 51, 50, and 55 cells). (*F*) Representative polysome profiles of HEK293 cells. (*G* and *H*) qRT-PCR analysis of *ABL2* and *RPS12* detected in sucrose gradient fractions from HEK293 cells (n = 3). (*I*) Scheme depicts the proposed model for AKAP12-mediated anchoring of mRNAs to the PM. Bars charts represent means ± SD. ns = nonsignificant, *P < 0.05, **P < 0.01.

In light of our findings, we hypothesized that AKAP12 may not only compartmentalize proteins but could also regulate subcellular mRNA localization. We used a density-based method to interrogate the presence of *ABL2* mRNA in PM fractions and whether it could be dependent on AKAP12. siRNA-mediated suppression of AKAP12 induced negligible changes in the cytosolic levels of *ABL2* mRNA and the nonbound *RPS12* mRNA (Fig. 2*A*). In contrast, AKAP12 suppression resulted in a remarkable reduction of *ABL2* mRNA present in PM fractions, without causing significant changes in *RPS12* mRNA levels (Fig. *2B*). Taking into account the functional importance of mRNA localization in protein synthesis (1), we sought to determine whether AKAP12 could regulate *ABL2* mRNA translation. Interestingly, proteomic studies carried out by Benz et al. (9) revealed that RNA binding proteins (RBPs) are coimmunoprecipitated with AKAP12 from EC extracts. These include the ribosomal subunits also detected within PM-rich EC protrusions (Fig. 2*C*), suggesting a close spatial relationship between AKAP12 and the translation machinery. Using antibodies targeting both proteins, we detected a robust proximity ligation assay (PLA) signal not observed in the absence of one or both antibodies (Fig. 2 *D* and *E*). To assess whether AKAP12 modulates *ABL2* mRNA translation, we performed polysome fractionation using material derived from siRNA-transfected cells. Down-regulating AKAP12 did not affect the polysome profiles (Fig. 2*F*), indicating seemingly undisturbed global translation levels. However, the relative amount of *ABL2* mRNA detected in polysome fractions was markedly reduced by the loss of AKAP12, despite unchanged overall levels in the prefractionation material (Fig. 2 *G* and *H*). In contrast, relative levels of polysome-associated *RPS12* mRNA remained unaltered (Fig. 2*H*), highlighting target-specific regulatory roles of AKAP12. Given the association of actively translating mRNAs with polysomes, these data indicate that AKAP12 is necessary for efficient *ABL2* mRNA translation.

## Discussion

The targeting of AKAP12 to the PM is tightly linked to its signal-induced regulation of the cytoskeleton and consequent PM remodeling, underpinning invasion and adhesion behaviors that are likely to be cell type and context dependent (10–12). Acting as a scaffold, AKAP12 compartmentalizes signaling effectors responsible for Actin reorganization in a multifaceted fashion. We showed that the nature of AKAP12-mediated anchoring targets in ECs is not limited to proteins but also extends to mRNAs. The fact that these include transcripts encoding Actin-binding proteins with cytoskeletal reorganization properties, prompts speculating that AKAP12-mediated distribution of mRNAs is mechanistically intertwined with and supports PM remodeling, with potential major implications in morphological phenomena such as angiogenesis (Fig. 2*I*).

Compounding evidence has established that subcellular mRNA localization can underpin local protein function. This includes several examples of RBP-mediated targeting of mRNAs to membrane compartments other than the PM, coupled to the localized synthesis of proteins that incorporate the compartment in question (13). Although not understood to the same extent, some reports have suggested that PM-linked translation may be a fundamental phenomenon. An elegant study by Winkenbach et al. demonstrated that the localization of the *C. elegans erm1* mRNA to the PM is mediated by the nascent membrane binding peptide in the encoded ERM1 as it emerges from the ribosome (14). Detailed analyses of EC surface proteomes have unveiled high abundancy of translation machinery components (15). Likewise, the identification of ribosomal proteins anchored to the intracellular domains of surface receptors in neurons hinted to the importance of protein synthesis in proximity with the PM (16). Although we are yet to fully demonstrate that AKAP12 targets are locally translated, we propose that PM-docked ribosomes could participate in the translation of mRNAs anchored by AKAP12 to this compartment. In summary, our work unveils a unique role of AKAP12 as a trans-acting factor that simultaneously localizes mRNAs and regulates protein synthesis.

## Methods

OOPS was carried out with cell protrusions formed in the underside of Transwell membranes. UV RIP was performed using cells exposed to 254 nm UV light, lysed, and incubated with an antibody targeting AKAP12. For two- and three-dimensional angiogenesis assays, ECs were cocultured with pulmonary fibroblasts. Gradient fractionation assays were carried out for the isolation of the PM and for polysome fractionation. Details of all methodologies applied in this study are included in *SI Appendix*.

## Supplementary Material

Appendix 01 (PDF)

Dataset S01 (XLSX)

Dataset S02 (XLSX)

Movie S1. Live imaging of siRNA-transfected ECs expressing Lifeact-GFP while cultured on a fibroblast monolayer. Scale bars, 20 μm.

## Data Availability

Mass spectrometry proteomics (17)and RNA-seq (18) data have been deposited in ProteomeXchange Consortium (via PRIDE: PXD046994; https://www.ebi.ac.uk/pride/) and Gene Expression Omnibus (GEO: GSE247682; https://www.ncbi.nlm.nih.gov/geo/), respectively.
