# Supplementary material for: The scaffolding protein AKAP12 regulates mRNA localization and translation
Source: Proc Natl Acad Sci U S A. 2024 Apr 23;121(18):e2320609121. doi: 10.1073/pnas.2320609121 (PMC11067055; doi:10.1073/pnas.2320609121)
Supplement: Supplementary file 1 — Appendix 01 (PDF) [file pnas.2320609121.sapp.pdf]

PNAS

Supporting Information for

The scaffolding protein AKAP12 regulates mRNA localization and translation.

Madeleine R. Smith, Parisa Naeli, Seyed M. Jafarnejad and Guilherme Costa*

*Guilherme Costa
Email: g.costa@qub.ac.uk

This PDF file includes:

- Supporting text
- Legends for Movies S1
- Legends for Datasets S1 to S2
- SI References

Other supporting materials for this manuscript include the following:

- Movies S1
- Datasets S1 to S2

Supporting Materials and Methods

Cell culture, transwell assays and transfections

Human umbilical vein endothelial cells (HUVECs; PromoCell) were routinely cultured on gelatine coated dishes in complete ECGM2 (PromoCell) containing 50 µg/ml gentamycin (Merck) and 50 ng/ml amphotericin (Merck). Human pulmonary fibroblasts (HPFs; PromoCell) and human embryonic kidney cells (HEK293) were routinely cultured in DMEM (Thermo Fisher Scientific) containing 10% FBS, 10 U/ml–100 µg/ml penicillin–streptomycin (Thermo Fisher Scientific).

Transwell assays were carried out as previously described(1). Cell bodies and protrusions were mechanically fractionated using cell scrapers, and the material washed into tubes with 20 µl extraction reagent per transwell for downstream analyses.

ON-TARGETplus siRNAs (Horizon) were transfected into HUVECs or HEK293 using GeneFECTOR (VennNova) or Lipofectamine 2000 transfection reagent (Thermo Fisher Scientific), respectively. HUVECs were electroporated with plasmid DNA using a HUVEC Nucleofector Kit (Lonza) in a Nucleofector™ 2b Device (Lonza) following manufacturer recommendations.

Coculture assays

HUVECs were co-cultured with HPFs as previously detailed by Hetheridge *et al.* (2). For filopodia tracking experiments, siRNA-treated HUVECs were harvested 3 hours post-transfection and electroporated with 1 µg LifeAct-GFP(3). Cells were then plated onto confluent HPFs and imaged 48 hours later.

Fibrin bead assay

The fibrin bead assay was adapted from Kempers *et al.*(4). Briefly, siRNA transfected HUVECs were harvested and incubated with Cytodex 3 microcarrier beads (Merck) for 4 hours, mixing every 20 minutes. The HUVEC-bead mixture was then transferred into T25 flasks and incubated overnight. Coated beads were then washed 3 times with ECGM2 and embedded in a fibrin gel (Merck) for 24 hours in a glass-bottomed 96 well plate. Sprouting beads were then washed with PBS before fixation with 4% formaldehyde for 15 minutes at room temperature. Beads were then permeabilized with PBS containing 0.5% Trition-X-100 for 5 minutes at 4 °C before washing with PBS and

staining with 1 µg/ml DAPI (Merck) and AlexaFluor™ 488 phalloidin (Thermo Fisher Scientific). Beads were washed 3 times before leaving in fresh PBS for imaging.

Orthogonal organic phase separation (OOPS) and liquid chromatography with tandem mass spectrometry (LC-MS/MS)

OOPS experiments followed the methodology developed by Queiroz *et al.*(5). Briefly, HUVECs cultured in 10 cm dishes or in Transwells were washed with ice cold PBS and crosslinked at 400 mJ/cm^2 with 254 nm UV light. Samples were collected in TRIzol (Thermo Fisher Scientific) and processed through 3 rounds of 5:1 TRIzol:chloroform mixing, 1,000 x *g* centrifugations for 15 minutes at 4 °C and removal of the aqueous and organic phases to purify the interphase. After the last round, the interphase was mixed with 9 vol. 100% methanol and RNA-protein adducts were precipitated by centrifugation at 14,000 x g for 10 minutes at room temperature. The pellets were resuspended in 50 µL of 1% SDS, 100 mM TEAB (Thermo Fisher Scientific) and 1 mM $MgCl_2$, heated for 20 minutes at 95 °C, cooled and then digested with 2 µg RNaseA/T1 mix (Thermo Fisher Scientific), firstly for 4 hours and then overnight at 37 °C with additional 2 µg RNaseA/T1 mix. To extract proteins, the samples were mixed with 5:1 TRIzol:chloroform, centrifuged at 12,000 x g for 15 minutes at 4 °C and fractions of the organic phase were sequentially processed through rounds of 9 vol. 100% methanol mixing followed by centrifugation at 14,000 x g for 10 minutes at room temperature. The final pellet was air dried and resuspended in Laemmli buffer (250 mM Tris–HCl pH 6.8, 2% SDS, 10% glycerol, 0.0025% bromophenol blue, 10% β-mercaptoethanol) before loading onto 10% Mini-PROTEAN® TGX™ precast protein gels (Bio-Rad). After short electrophoresis, protein bands were stained with InstantBlue (Expedeon), excised from the gel and dehydrated using acetonitrile followed by vacuum centrifugation. Dried gel pieces were reduced with 10 mM dithiothreitol and alkylated with 55 mM iodoacetamide. Gel pieces were then washed alternately with 25 mM ammonium bicarbonate followed by acetonitrile. This was repeated, and the gel pieces dried by vacuum centrifugation. Samples were digested with trypsin overnight at 37 °C and analyzed by LC-MS/MS using an UltiMate® 3000 Rapid Separation LC (RSLC, Dionex Corporation) coupled to an Orbitrap Elite (Thermo Fisher Scientific) mass spectrometer. Peptide mixtures were separated using a gradient from 92% A (0.1% FA in water) and 8% B (0.1% FA in acetonitrile) to 33% B, in 44 min at 300 nL min^{-1}, using a 75 mm x 250 µm i.d. 1.7 mM BEH C18, analytical column (Waters). Peptides were selected for fragmentation automatically by data

dependent analysis. Data produced were searched using Mascot (Matrix Science), against the Swissprot database with taxonomy of *Homo sapiens* selected. Data were validated using Scaffold (Proteome Software).

UV RNA immunoprecipitation (RIP) and RNA sequencing

UV RIP was performed as described in Holmes *et al.*(6). HUVECs were washed with ice cold PBS and crosslinked at 400 mJ/cm^2 with 254 nm UV light. Cells were then lysed in Pierce IP lysis buffer (Thermo Fisher Scientific) containing 0.1 U/µL RNAseOut (Thermo Fisher Scientific) and 1× HALT PIC (Thermo Fisher Scientific), rotated for 10 minutes at 4 °C and centrifuged at max speed for 10 minutes at 4 °C. Lysates were then precleared with Dynabeads Protein G (Thermo Fisher Scientific) for 45 minutes at 4 °C with rotation before incubation with 2 mg/ml anti-AKAP12 (Sigma) or 2 mg/ml mouse IgG (Millipore), antibodies overnight at 4 °C. Samples were then incubated with Dynabeads for 1 hour at 4°C before 3 washes with wash buffer (25 mM Tris pH 7.5, 150 mM KCl, 0.5% NP-40, 5 mM EDTA, 0.5 mM DTT, 0.1 U/µL RNAseOut, 1× HALT PIC). Samples were incubated with reverse-crosslinking buffer (1× PBS, 4% N-lauroyl sarcosine, 20 mM EDTA, 10 mM DTT, 2 U/µL RNAseOUT, 2.4 µg/µL Prot K) in the thermomixer at 42 °C for 1 hour and 53 °C for 1 hour at 400 rpm. RNA was then isolated with TRIzol and chloroform, and precipitated with 1 vol. of isopropanol, 0.1 vol. of sodium acetate and 1 µL of GlycoBlue Coprecipitant (Thermo Fisher Scientific) overnight at -80°C. The samples were centrifuged at maximum speed, the pellets washed with 70% ethanol and resuspended in RNAse free water.

For RNA sequencing, quality and integrity of RNA samples obtained from UV RIP experiments were analyzed using a TapeStation (Agilent Technologies). Next, libraries were generated using the KAPA RNA HyperPrep Kit with RiboErase (Kapa Biosystems) according to the manufacturer's protocol. Library QC was carried out in a MiSeq system (Illumina) and a loaded flow cell was then paired-end sequenced on an NextSeq 2000 instrument (Illumina). Processed reads were aligned to the reference human genome (Release 33 from GRCh38.p13 build) using STAR 2.7.3a(7). Count assignment was performed with HTSeq –count using annotation from GENCODE (http://www.gencodegenes.org/), and post-alignment QC with Picard and Qualimap, and MultiQC version 1.7 combining all QC. Enrichment of RIP samples over input samples was calculated with Bioconductor package DESeq2 v1.42.0(8) and visualized with EnhancedVolcano(9). Enriched transcripts were selected filtering DESeq2 results based

on fold change ($log_2 > 1$) and adjusted p value ($p < 0.01$). The resulting transcript list was processed through Gene Ontology analysis using the Database for Annotation, Visualization and Integrated Discovery (DAVID)(10, 11). *ABL2* was chosen for downstream studies due to its *actin filament binding* and *kinase* activities.

RNA isolation, cDNA Synthesis and quantitative (q)RT-PCR

RNA was isolated using the RNeasy Plus Micro Kit (Qiagen) following the manufacturer's protocol and analyzed at 254 nm using a NanoDrop One instrument (Thermo Fisher Scientific). cDNA was synthesized using the High-Capacity RNA-to-cDNA Kit (Thermo Fisher Scientific) according to the manufacturer's protocol. qRT-PCR experiments were prepared using LightCycler 480 SYBR Green I Master (Roche) and target specific primers (Merck): ***AKAP12*** F 5'-CCCAAGCACAGGAGGAGTTA-3', ***AKAP12*** R 5'- TGCCTGCTCTCCAATTCTCA-3', ***ABL2*** F 5'-GTCCATCTCGCTCAGGTACG-3', ***ABL2*** R 5'- TGTTGTCACCAGCCCATCAG-3', ***RPS12*** F 5'- AGCCCATCTTTGTGTGCTTG-3', ***RPS12*** R 5'-TGCAACCAACCACTTTACGG-3', ***Luciferase*** F 5'- GAGCACGGAAAGACGATGACGG-3', ***RPS12*** R 5'- GGCCTTTATGAGGATCTCTCTG-3'. Reactions were carried out in triplicate technical replicates in 384 well plates using a Lightcycler 480 instrument (Roche). Analysis was then performed using the $2^{-\Delta\Delta CT}$ method to compare relative mRNA levels normalized to input samples or Luciferase mRNA.

RNA extraction from plasma membrane

siRNA-transfected cells were washed with ice cold PBS and crosslinked at 400 mJ/cm^2 with 254 nm UV light. Cells were then scraped in PBS and centrifuged at 1500 rpm for 5 minutes and processed using the Plasma Membrane Protein Extraction Kit (Abcam) with the following modifications. Pellets were resuspended in homogenization buffer containing 0.1 U/µL RNAseOut (Thermo Fisher Scientific) and homogenized 40 times using a 26 G needle and 1 mL syringe. Homogenized samples were centrifuged in 700 x *g* for 10 minutes at 4 °C and the supernatant centrifuged at 10,000 x *g* for 30 minutes at 4 °C to yield the cytosolic fraction (supernatant) and the total cellular membrane proteins (pellet). The total membrane proteins pellet then underwent phase separation with centrifugations in 1,000 x *g* for 5 minutes at 4 °C. The upper aqueous phase was diluted in 5 volumes of RNAse free H_2O, incubated for 5 minutes on ice and centrifuged at maximum speed for 10 minutes at 4 °C. The resulting plasma membrane protein pellet

was resuspended in PBS containing 0.5% Triton X-100. Protein in both the cytosolic and plasma membrane fractions were quantified using the Pierce BCA Protein Assay Kit (Thermo Fisher Scientific), following manufacturer's recommendations. Equal amounts of each fraction were then incubated with reverse-crosslinking buffer (2× PBS for Cytosolic Fraction, 1× PBS for Plasma Membrane fraction, 4% N-lauroyl sarcosine, 20 mM EDTA, 10 mM DTT, 2 U/µL RNAseOUT, 2.4 µg/µL Prot K) in a thermomixer at 42 °C for 1 hour and 53 °C for 1 hour at 400 rpm. RNA extraction, precipitation and reconstitution steps were carried out following the corresponding methodology described in the UV RIP section.

Proximity ligation assay (PLA)

PLA was carried with Duolink PLA reagents (Merck) and following the manufacturer's protocol. Briefly, cells were cultured on glass coverslips, fixed with 4% formaldehyde before permeabilising in PBS containing 0.5% Triton X-100 and blocking in 4% goat serum. Coverslips were incubated with primary antibodies in blocking solution for 1.5 hours at room temperature – 1:500 mouse AKAP12 (Sigma) and 1:100 rabbit RPL13 (Abcam) – and next with rabbit PLUS and mouse MINUS probes. Ligation and amplification steps were performed with the Duolink Far Red Detection kit. Coverslips were then counterstained with both 1 µg/ml DAPI and AlexaFluor™ 488 phalloidin, and finally mounted with ProLong Gold Antifade Mountant.

Polysome profiling

siRNA-transfected cells were pretreated with 100 µg/ml cycloheximide (Sigma) for 5 minutes before collection by centrifugation at 1,200 x g for 5 minutes at 4 °C. Cells were then lysed in 500 µl hypotonic buffer (5 mM Tris-HCl pH 7.5, 2.5 mM $MgCl_2$, 1.5 mM KCl, complete EDTA-free protease inhibitor tablet, 100 U RNAseOUT (Thermo Fisher Scientific), 100 µg/ml cycloheximide, 2 mM dithiothreitol, 0.5% v/w Triton X-100 and 0.5% v/w sodium deoxycholate). Lysates were cleared through centrifuged at 20,000 x g for 5 minutes at 4 °C and the total RNA concentration of each sample was measured at 254 nm using a NanoDrop One instrument. 90 µg RNA were loaded and separated on 14 ml of a 10-50% sucrose gradient by ultracentrifugation at 230,500 x g for 2 hours at 4 °C using a SW40 rotor (Beckman Coulter). Absorbance at 254 nm was measured from lower to higher sucrose gradients using an ISCO gradient fractionation system and the optical density at 254 nm was continuously recorded with a Foxy JR Fractionator

(Teledyne ISCO). Fractions were spiked with 250 ng Luciferase Control RNA (Promega) before adding TRIzol and incubated overnight at -80 °C. RNA extraction, precipitation and reconstitution steps were carried out following the corresponding methodology described in the UV RIP section.

Microscopy and computational analysis

Live imaging of endothelial cells for filopodia was performed in a Nikon 6D (Eclipse Ti-E) inverted Microscope equipped with a Neo 5.5 sCMOS ANDOR Camera with 5.5MP and 2560x2160 16.6 mm x 14.0 mm sensor size, using a 100x/1.45/NA/CFI60 Plan Apochromat Lambda oil objective and NIS-A Elements software. Cells were imaged every 5 seconds for 5 minutes. Filopodia analysis was performed using the Filopodyan ImageJ plugin and R scripts developed by Urbančič *et al.*(12).

Microscopy images of fixed samples were acquired with a Leica STELLARIS 5 LiA Confocal Microscope (using either 10x/0.4NA/HC/PL/PO/CS2 or 100x/1.4/NA/HC/PL/APO/CS2/Oil objectives).

Statistical analysis

Data were analyzed using GraphPad Prism 9 (GraphPad Software) and results are expressed as means ± standard deviation. D'Agostino–Pearson or Shapiro–Wilk tests were carried out to analyze the normal distribution of the variables ($P>0.05$). Comparisons of two groups were assessed using paired t-tests. One-way analysis of variance (ANOVA) with a Tukey’s post-hoc test was used to analyze three of more groups. For three or more groups of nonparametric data, a Kruskal-Wallis test with Dunns post-hoc analysis was performed. Statistical significance is reported for $P<0.05$.

Movie S1 (separate file). Live imaging of siRNA-transfected ECs expressing Lifeact-GFP while cultured on a fibroblast monolayer. Scale bars, 20 μm.

Dataset S1 (separate file). RNA-bound proteins identified via OOPS in EC protrusions and whole cell samples.

Dataset S2 (separate file). Transcripts identified in AKAP12 UV RIP-seq experiments.

SI References

1. G. Costa, J. J. Bradbury, N. Tarannum, S. P. Herbert, RAB13 mRNA compartmentalisation spatially orients tissue morphogenesis. *EMBO J* **39**, e106003 (2020).
2. C. Hetheridge, G. Mavria, H. Mellor, Uses of the in vitro endothelial-fibroblast organotypic co-culture assay in angiogenesis research. *Biochem Soc Trans* **39**, 1597-1600 (2011).
3. B. J. Belin, L. M. Goins, R. D. Mullins, Comparative analysis of tools for live cell imaging of actin network architecture. *Bioarchitecture* **4**, 189-202 (2014).
4. L. Kempers, I. van der Bijl, A. D. van Stalborch, B. Ponsioen, C. Margadant, Fast in vitro protocol for the visualization and quantitative high-throughput analysis of sprouting angiogenesis by confocal microscopy. *STAR Protoc* **2**, 100690 (2021).
5. R. M. L. Queiroz *et al.*, Comprehensive identification of RNA-protein interactions in any organism using orthogonal organic phase separation (OOPS). *Nat Biotechnol* **37**, 169-178 (2019).
6. Z. E. Holmes *et al.*, The Sox2 transcription factor binds RNA. *Nat Commun* **11**, 1805 (2020).
7. A. Dobin *et al.*, STAR: ultrafast universal RNA-seq aligner. *Bioinformatics* **29**, 15-21 (2013).
8. M. I. Love, W. Huber, S. Anders, Moderated estimation of fold change and dispersion for RNA-seq data with DESeq2. *Genome Biol* **15**, 550 (2014).
9. R. S. Blighe K., Lewis M. (2019) EnhancedVolcano: publication-ready volcano plots with enhanced colouring and labeling.
10. W. Huang da, B. T. Sherman, R. A. Lempicki, Systematic and integrative analysis of large gene lists using DAVID bioinformatics resources. *Nat Protoc* **4**, 44-57 (2009).
11. B. T. Sherman *et al.*, DAVID: a web server for functional enrichment analysis and functional annotation of gene lists (2021 update). *Nucleic Acids Res* **50**, W216-W221 (2022).
12. V. Urbancic *et al.*, Filopodyan: An open-source pipeline for the analysis of filopodia. *J Cell Biol* **216**, 3405-3422 (2017).
